# Terazosin Treatment Induces Caspase-3 Expression in the Rat Ventral Prostate

**DOI:** 10.4021/jocmr1215w

**Published:** 2013-02-25

**Authors:** Georgios Papadopoulos, Dimitrios Vlachodimitropoulos, Aspasia Kyroudi, Mirsini Kouloukoussa, Despina Perrea, Dionisios Mitropoulos

**Affiliations:** a Department of Histology and Embryology, University of Athens Medical School, Greece; b Department of Experimental Surgery and Surgical Research, University of Athens, Medical School, Greece; c 1st Department of Urology, University of Athens Medical School, Greece

**Keywords:** Alpha-adrenoreceptor antagonists, Terazosin, Caspase-3, Apoptosis, Rat, Prostate

## Abstract

**Background:**

Quinazoline-based alpha1-adrenergic receptor antagonists may not act solely on smooth muscle contractility. We evaluated the in vivo effect of terazosin on the expression of caspase-3 in the rat ventral prostate.

**Methods:**

Fifteen Wistar rats were treated with terazosin (1.2 mg/kg body weight, given orally every second day) for 120 days. Another 15 control animals received the same amount of distilled water. The expression of caspase-3 was assessed immunohistochemically in formalin-fixed, paraffin-embedded tissue sections.

**Results:**

Terazosin treatment did not affect prostate weight and histomorphology. In controls caspase-3 was expressed weakly and sporadically. In contrast, strong and weak expression was evident in 67% and 33% of the terazosin-treated specimens, respectively.

**Conclusions:**

These findings implicate the induction of caspase-3 expression by terazosin as a potential molecular mechanism of its apoptotic action on prostate cells.

## Introduction

The rationale for using α1-adrenergic receptor (α1-ADR) antagonists for the treatment of patients with benign prostate hyperplasia (BPH)-related lower urinary tract symptoms stems from the fact that they effectively relax prostate smooth muscles, which represent approximately 40% of the cellular volume in hyperplastic glands [1]. Moreover, it is widely accepted that catecholamines, besides affecting secretory function, have a direct mitogenic effect on prostate growth, acting directly through the adrenergic receptors or through receptor-mediated induction of other growth factors [2]. Rapid proliferation of prostatic epithelial cells has also been seen in the spontaneously hypertensive rat [3], an animal model with increased prostatic norepinephrine levels, while prostatic stromal cell phenotype is directly modulated by norepinephrine [4].

An accumulation of data from several studies indicates that quinazoline-based α1-ADR antagonists may not act solely on smooth muscle contractility. Treatment of cell cultures with doxazosin inhibited the differentiation of prostatic stromal cells towards a differential smooth muscle phenotype [5]. This was also observed after in vivo α1-ADR blockade in either man or rats which resulted in significant decrease of smooth muscle myosin heavy chain gene expression [6]. Prostatic cell apoptosis has been identified as an additional mechanism of long term action for doxazosin and terazosin [7, 8], while it has been postulated that the apoptotic effect is probably quinazoline nucleus directed rather than α1-ADR mediated [9]. The apoptotic effect of α1-ADR antagonists has been attributed to transforming growth factor-β1 (TGF-β1) since in vitro treatment of primary human prostate cell cultures with doxazosin [10] as well as in vivo treatment with terazosin [11] resulted in significantly enhanced TGF-β1 expression. Consequently, this results in upregulation of p27^kip-1^, a downstream intracellular effector of TGF-β1 apoptotic signaling and, possibly, activation of the caspase cascade [12].

In previous studies we have showed that terazosin may also exert its apoptotic action through a differential effect on the glycosaminoglycans and matrix metalloproteinase 2 of the rat prostate extracellular matrix (ECM) [13], and by inducing a considerable decrease in basic fibroblast growth factor (bFGF) immunohistochemical expression [14]. ECM regulates, among others, the trafficking of growth factors, while bFGF is one of the main promoters of cellular proliferation inducing epithelial growth [15]. Herein, we further explored the apoptotic consequences of terazosin and report its effect on caspase-3 expression.

## Materials and Methods

Wistar rats were housed in a climatized environment (temperature 21 °C, humidity 55 ± 5%) with 12-hour light/dark cycle. Rats were fed a standard laboratory diet and water ad libidum. Principles of laboratory animal care were followed and the study complied with European Union regulations for the care and use of laboratory animals. Body weight was determined once a week.

### Study design

Thirty 100 ± 5 day-old male rats were randomly allocated into two groups. One group (15 rats) received treatment with terazosin hydrochloride (Abbott Laboratories) (1.2 mg/kg body weight every second day) dissolved in water for injection. The maximum recommended human daily dose is 20 mg. Toxic effects are not anticipated from the dose we used, since terazosin LD_50_ in rats following oral administration ranges from 5.5 to 6.6 g/kg, while the “no-toxic-effect” dosage and the maximum-tolerated dose are 60 mg/kg/day and 150 mg/kg/day, respectively (Abbott Laboratories, data on file). Treatment was given through a specially modified oesophageal catheter for 120 days. Fifteen control animals received the same amount of distilled water. At the end of the experiment, rats were killed with an overdose of ethyl ether, and the ventral prostate glands were dissected, weighted, fixed in 10% buffered formalin and embedded in paraffin. T-test was the statistical method that was used.

### Immunohistochemistry

Immunohistochemistry for caspase-3 was performed on formalin-fixed, paraffin-embedded 5 to 6 μm thick tissue sections. The sections were mounted on positively charged glass slides (Superfrost Plus; Menzel-Glaser, Germany), deparaffinized in xylene and rehydrated with graded alcohols. Consequently, they were treated for 20 min at 98 °C in 0.01 M citric buffer (pH 6.0), rinsed in osmosed water (twice for 5 min each), and washed (twice for 5 min each) in Tris-buffered saline (TBS). After applying the secondary antibody (Santa Cruz Biotechnology, Inc., Santa Cruz, CA, USA) and incubation for 30 min at room temperature, reactivity was visualized with an avidin-biotin complex peroxidase system (Santa Cruz Biotechnology, Inc., Santa Cruz, CA, USA). Non-immune normal IgG at equivalent dilutions to the primary antibodies were used as negative controls. The slides were counterstained with hematoxylin and reviewed; fifty high power fields were reviewed in each slide. Immunoreactivity was characterized as absent, weak or strong while positivity was expressed as the percentage of stained cells over the total number of cells. Statistical analysis was performed by using t-test.

## Results

All rats treated with either terazosin or sterile water remained healthy throughout the course of the treatment and showed the expected continuous increase in their body weights as previous studies described [16]. At sacrifice, body weight as well absolute and relative (defined as absolute prostate weight to body weight) ventral prostate wet weights were not affected by terazosin treatment (P > 0.1) (Table 1). Moreover, terazosin treatment did not result in any macroscopic or microscopic alterations in prostate morphology (P > 0.1).

**Table 1:** Body Weight (BW), Absolute Prostate Weight (APW), and Relative Prostate Weight (RPW) of Terazosin-Treated (Group A) and Control (Group B) Rats (Mean ± Standard Deviation)

	BW (g)		APW (g)	RPW (mg/g)
	start	end		
Group A	430 ± 39	459 ± 42	0.61 ± 0.11	1.28 ± 0.29
Group B	418 ± 19	490 ± 27	0.52 ± 0.02	1.07 ± 0.05

**Figure 1:**
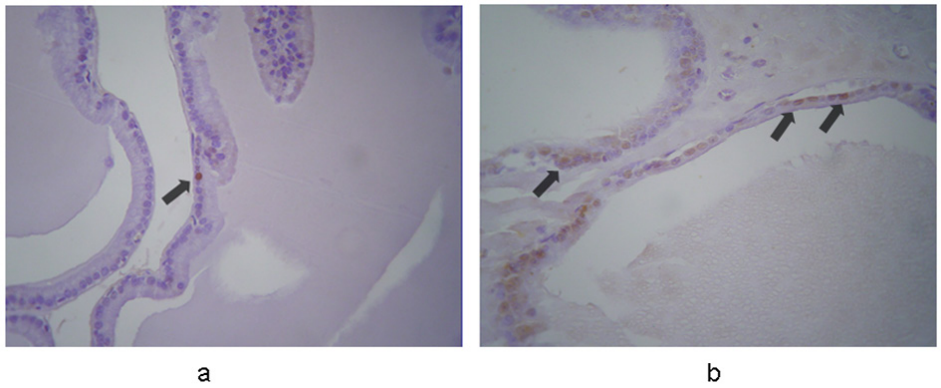
Immunohistochemical expression of caspase-3 in the prostates from (a) control and (b) terazosin-treated rats (magnification × 100). In controls caspase-3 is expressed in epithelial cells weakly and sporadically. In contrast, in terazosin-treated specimens, stronger expression is evident in a significantly larger proportion of epithelial cells, mainly in those detached to the lumen of the acini. The arrows show positive nuclei in caspase-3 expression.

In controls, caspase-3 was expressed weakly and sporadically in 0.7-1.7% of cells (mean, 1.1%). In contrast, strong and weak expression was evident in 67% and 33% of the terazosin-treated specimens, respectively. Cytoplasmic immunoreactivity was observed in 6.7-8.9% (mean, 8%) of epithelial cells, mainly in those detached to the lumen of the acini (Fig. 1). This indicated statistically significant difference between the two groups (P < 0.001).

## Discussion

The objective of this study was to further explore the mechanisms underlying the apoptotic effect of terazosin, a commonly used quinazoline-based α1-ADR antagonist, on prostatic cells by studying the immunohistochemical expression of caspase-3 in the rat ventral prostate following a period of in vivo treatment. The results indicate that systemic terazosin treatment for 4 months did not affect prostatic growth and morphology as indicated by the similar wet prostate weights and microscopic appearance. As far as prostate volume is concerned, terazosin administration in humans resulted in the same mean change from baseline observed in the placebo group [17]. In a mouse model of prostatic hyperplasia, administration of doxazosin in dosages well above those used to achieve complete blockade of α1-ADRs in rodents resulted in significant reduction in the wet weight of prostate reconstitutions with retroviral transduction of TGF-β1 [7]. In this model, weight reduction was attributed to increased apoptosis induced by doxazosin treatment. Conversely, exposure of spontaneously hypertensive rats to doxazosin did not result in reduction of the glandular epithelium volume and rather contributed to protecting against caspase-induced apoptosis [18]. Both doxazosin and terazosin have proven their in vivo effect on hyperplastic prostate tissue in humans, by inducing cell apoptosis with microscopically apparent severe stroma degeneration and collagen accumulation [8]. We could not observe any changes in histomorphology, but this may represent a different reaction of normal rat tissue than that of human prostatic hyperplasia. Support on this notion stems from the observations in the mouse model, in which doxazosin treatment induced apoptosis in prostate reconstitutions, but did not affect the glandular epithelia of the ventral prostate of the engrafted mice [7].

Under normal conditions, prostate cell apoptosis may be interpreted as a default mechanism or a dormant process requiring direct activation [19]. Caspases are central to the induction of the apoptotic cascade leading to controlled cell death [20] and, based on the size of their prodomains, they can be functionally classified into two groups: those with longer prodomains (caspases 2, 8 and 10) that are implicated in targeting and regulating activation, and those with shorter prodomains (caspases 3, 6, 7 and 9) that function more downstream in the apoptotic pathway, cleaving critical substrates [21]. Activation of the caspase cascade mediates apoptosis in prostate cancer cells [22]. Furthermore, caspases-3, -6 and -9 are activated during finasteride treatment of benign prostatic hyperplasia [23].

Immunohistochemical analysis has shown expression of pro-caspase-3 in normal secretory epithelial cells [24], and in benign prostatic hyperplasia and prostatic carcinoma [25]. Treatment of prostate cancer cells with doxazosin resulted in a strong caspase-3 activation within 24 h, while tamsulosin had no similar effect [26]. This supports the notion that quinazoline derivatives activate apoptosis through TGF-β1 signaling since TGF-β1 has been shown to activate caspase-3 to induce apoptosis in the NRP-154 prostate epithelial cell line [27]. However, data opposing the activation of the TGF-β transduction pathway being responsible for quinazoline-derived α1-ADR antagonists-induced apoptosis have been recently published [28]. Instead, molecular targets consistent with tumor necrosis factor (TNF)-α-related activity were identified [28, 29]. Indeed, TNF-α induces cell death and cell proliferation within prostate tissues [28, 29]. Nevertheless, caspase-3 is a crucial enzyme for the executive phase of apoptosis that can be activated after the stimulation of TNF family receptors; doxazosin may influence TNF receptor expression without affecting their functional status [28].

Quinazoline-derived α1-ADR antagonists have been also shown to induce anoikis (the apoptosis induced by the loss of cell attachment to the ECM) in prostate cancer cells; anoikis resistance may arise from loss of apoptotic signaling via inhibition of caspase activity [30]. Our previous observations on the differential effect of terazosin on the glycosaminoglycans and matrix metalloproteinase 2 of the rat prostate ECM [13], provides further support the hypothesis of a complex molecular cross-talk of the cell death actions induced by quinazolines.

### Conclusions

Our study provides evidence to suggest that induction of caspase-3 expression following terazosin treatment in vivo could be one of the underlying molecular mechanisms contributing to the overall action and long-term efficacy of this drug. Furthermore, it provides further insight into the ability of quinazolines to affect prostate homeostasis by interfering with cellular apoptosis rather than proliferation.
